# Predictive value of the domain specific PLA2R antibodies for clinical remission in patients with primary membranous nephropathy: A retrospective study

**DOI:** 10.1371/journal.pone.0302100

**Published:** 2024-05-08

**Authors:** Kezhi Zhou, Junyi Zhou, Leting Zhou, Jing Xue, Bin Liu, Zhijian Zhang, Xiran Zhang, Ting Cai, Sijia Shao, Biao Huang, Yi Zhang, Zhigang Hu, Liang Wang, Xiaobin Liu

**Affiliations:** 1 Department of Nephrology, The Affiliated Wuxi People’s Hospital of Nanjing Medical University, Wuxi People’s Hospital, Wuxi Medical Center, Nanjing Medical University, Wuxi, P.R. China; 2 College of Life Sciences and Medicine, Zhejiang Sci-Tech University, Hangzhou, P.R. China; 3 NHC Key Laboratory of Nuclear Medicine, Jiangsu Key Laboratory of Molecular Nuclear Medicine, Jiangsu Institute of Nuclear Medicine, Wuxi, P.R. China; 4 Medical Laboratory, The Affiliated Wuxi Children’s Hospital of Nanjing Medical University, Wuxi, P.R. China; The University of the West Indies, JAMAICA

## Abstract

**Background:**

M-type phospholipase A2 receptor (PLA2R) is a major auto-antigen of primary membranous nephropathy(PMN). Anti-PLA2R antibody levels are closely associated with disease severity and therapeutic effectiveness. Analysis of PLA2R antigen epitope reactivity may have a greater predictive value for remission compared with total PLA2R-antibody level. This study aims to elucidate the relationship between domain-specific antibody levels and clinical outcomes of PMN.

**Methods:**

This retrospective analysis included 87 patients with PLA2R-associated PMN. Among them, 40 and 47 were treated with rituximab (RTX) and cyclophosphamide (CTX) regimen, respectively. The quantitative detection of -immunoglobulin G (IgG)/-IgG4 targeting PLA2R and its epitope levels in the serum of patients with PMN were obtained through time-resolved fluorescence immunoassays and served as biomarkers in evaluating the treatment effectiveness. A predictive PMN remission possibility nomogram was developed using multivariate logistic regression analysis. Discrimination in the prediction model was assessed using the area under the receiver operating characteristic curve (AUC-ROC).Bootstrap ROC was used to evaluate the performance of the prediction model.

**Results:**

After a 6-month treatment period, the remission rates of proteinuria, including complete remission and partial remission in the RTX and CTX groups, were 70% and 70.21% (P = 0.983), respectively. However, there was a significant difference in immunological remission in the PLA2R-IgG4 between the RTX and CTX groups (21.43% vs. 61.90%, P = 0.019). Furthermore, we found differences in PLA2R-CysR-IgG4(P = 0.030), PLA2R-CTLD1-IgG4(P = 0.005), PLA2R-CTLD678-IgG4(P = 0.003), and epitope spreading (P = 0.023) between responders and non-responders in the CTX group. Multivariate logistic analysis showed that higher levels of urinary protein (odds ratio [OR], 0.49; 95% confidence interval [CI], 0.26–0.95; P = 0.035) and higher levels of PLA2R-CTLD1-IgG4 (OR, 0.79; 95%CI,0.62–0.99; P = 0.041) were independent risk factors for early remission. A multivariate model for estimating the possibility of early remission in patients with PMN is presented as a nomogram. The AUC-ROC of our model was 0.721 (95%CI, 0.601–0.840), in consistency with the results obtained with internal validation, for which the AUC-ROC was 0.711 (95%CI, 0.587–0.824), thus, demonstrating robustness.

**Conclusions:**

Cyclophosphamide can induce immunological remission earlier than rituximab at the span of 6 months. The PLA2R-CTLD1-IgG4 has a better predict value than total PLA2R-IgG for remission of proteinuria at the 6^th^ month.

## Introduction

Membranous nephropathy (MN) is the main type of adult nephrotic syndrome, which is caused by binding of auto-antibodies to antigens expressed on the podocyte surface. These auto-antibodies are directed against PLA2R and other antigens in 70–80% and 10–15% of cases, respectively [1–3]. As most cases of MN are associated with PLA2R, the total anti-PLA2R antibody levels are useful for disease risk stratification, treatment response, and recurrence [4, 5]. However, cases that call for additional biomarkers still exist. Most patients with high anti-PLA2R antibody titers have poor renal outcomes [4, 6, 7], but some exhibit different disease prognosis. Furthermore, although PLA2R is highly expressed in the kidney podocytes [1], alveolar type II epithelial cells [8], and neutrophils [9], anti-PLA2R autoantibodies only induce autoimmune diseases limited to the kidney. These factors indicate the importance of researching conformational antigenic epitopes of PLA2R.Further research the clinic role of these antigen epitopes can help to refine patients subgroups and explain diverse disease outcomes.

In the last decade, various immune-dominant epitopes of the PLA2R antigen, including the CysR, CTLD1, and CTLD7 domains, have been reported [10–12]. In 2016, Polski et al. found that proteinuria remission rate may be associated with the number of epitope domains involved in the immune reaction in patients with PMN, leading to the hypothesis of ‘epitope spreading’. The GEMRITUX study demonstrated that epitope spreading at baseline was an independent risk factor for adverse prognosis [13]. However, different perspectives on the role of epitope spreading exist. Reinhard et al. observed that clinical outcomes were related to total anti-PLA2R antibody levels, but not the epitope-recognition profiles at the time of diagnosis [14]. The research recommendations in the 2021 Kidney Disease Improving Global Outcomes (KDIGO) guideline note the importance of understanding the mechanisms of epitope spreading and immunodominance and determining whether analysis of epitope reactivity has a predictive value greater than that of PLA2R-antibody level [15].

Concerning the treatment of PMN, according to the 2021 KDIGO guideline, immunosuppressive therapy should be based on the disease risk stratification. In detail, rituximab(RTX) is the first-line therapy, and cyclophosphamide (CTX) remains the first choice for patients at a very high risk [15]. However, the precise treatment for high risk patients is still controversial. Epitope spreading has been reported to be associated with a decreased remission rate at six months [13, 16]. Also, the percentage of epitope spreaders tends to be lower after 6 months of RTX [13, 17]. It merits additional study to determine if it improves outcomes compared with those in patients who fail to experience spreading reversal.

Based on our previous studies [18–21], we have discovered the clinical significance of PLA2R-IgG and -IgG4, using time-resolved fluorescence immunoassays (TRFIA), for disease diagnosis, risk stratification, and prediction of proteinuria remission. Furthermore, we have established the TRFIA method to detect specific domain antibodies targeting PLA2R antigen in 2022 [22]. In this study, we retrospectively analyzed the efficacy of both RTX and CTX in combination with steroids in the treatment of PLA2R-associated PMN. We also aimed to evaluate the clinical value of the PLA2R domain-specific antibodies and epitope spreading as sensitive laboratory biomarkers for proteinuria remission and prognostic prediction.

## Materials and methods

### Study cohort

From January 2020 to August 2022, a total of 97 patients were screened. Ten had received calcineurin inhibitors and were excluded to prevent statistical bias. The remaining 87 consecutive patients treated at Affiliated Wuxi People’s Hospital of Nanjing Medical University were retrospectively reviewed and met the following criteria: (i) had biopsy-proven PLA2R-associated PMN; (ii) accepted RTX or CTX regimen after serum sample collection; and (iii) had no chronic infectious diseases that affect immunosuppressive therapy, such as tuberculosis. All renal tissue specimens were examined using light microscope, immunofluorescence, and electron microscope with routinely PLA2R and IgG subclass staining. Pathological grading was performed by Ehrenreich and Churg standards. Clinical data were extracted from the patients’ medical records. All enrolled patients provided written informed consent for their participation in the study. The study protocol was approved by the Institutional Review Board of the Affiliated Wuxi People’s Hospital of Nanjing Medical University (ethical approval no. kyl2016001). Additionally, access to information that could potentially identify individual participants post data collection was secured.

### Patient sera

Blood samples were collected before the immunosuppressive therapy and after 3, 6, and 12 months of treatment and were stored at −80°C after centrifuging at 3,000 rpm/min for 5 min and tested in batches in our laboratory.

### Interventions and follow-up

Risk stratification and choice of therapeutic regimens were performed in accordance with the 2021 KDIGO guidelines or its draft after 2020. After diagnosis, all patients received renal-support therapy using angiotensin-converting enzyme inhibitors or angiotensin receptor blockade. The doses administered ranged from normal dose to the maximally tolerated dose.

Patients who were assigned to the RTX group received 1,000 mg of intravenous medication on days 1 and 15. A second course of RTX was administered if the patient did not achieve complete remission or if the CD19+ B-cell count reached >5 cells/mL at 6 months. Patients who were assigned to the CTX group received weight-adjusted intravenous CTX once a month, combined with oral dose-adjusted prednisone(0.6−1.0 g/m^2^).

All patients underwent a series of follow-up appointments after the initiation of treatment at 0, 3, 6, and 12 months, and, then, at six-month intervals until reaching our endpoints. The following laboratory evaluations were performed at baseline and at every visit: 24-hour proteinuria, serum albumin, creatinine, estimated glomerular filtration rate (eGFR), anti-PLA2R antibody, and epitope levels.

### Endpoints and definitions

The endpoint was the composite of complete or partial remission at our follow-up. Complete remission(CR) was defined as proteinuria<0.3g/24h, and partial remission was defined as proteinuria <3.5g/24h or a reduction of >50% from baseline, with improvement or normalization of serum albumin concentration, and stable or elevated <30% from baseline of serum creatinine [23].

### Detection of antibodies for PLA2R and its epitopes by TRFIA

The purified PLA2R and domain proteins (CysR, CTLD1, and CTLD678) were coated in 96-well plates (2 μg/mL, 100 μL/well). Then, we added diluted serum samples in quadruplicate to the antigen-coated plates and placed them on the incubator shaker at 25°C for 1 h. After washing three times with wash buffer, Eu3+-labeled anti-human IgG/G4 antibody (diluted 1:100 in assay buffer) was added (100 μL/well) in the plates, which were managed as mentioned before. After rinsing for six times, 200 μL enhancement solution was added to the 96-well plates and the plates were agitated for 5 min. Finally, a TRFIA analyzer (excitation and emission wavelengths were 340 and 613 nm, respectively) was used to measure fluorescence. More specific details are available in a previous study [22].

Twenty sera from healthy volunteers were used to define the normal range by using mean±3SD as the cut-off value. The cut-off values of serum PLA2R-IgG/G4 were 13.23RU/mL and 66.66RU/mL, respectively. The cutoff values of serum PLA2R-CysR/CTLD1/CTLD678-IgG were 10.02RU/mL,16.01RU/mL and 14.38RU/mL, respectively. The cutoff values of serum PLA2R-CysR/CTLD1/CTLD678-IgG4 were 153.87RU/mL,37.66RU/mL and 126.91RU/mL, respectively.

### Epitope spreading

Epitope spreading was defined as PLA2R-CysR being the primary dominant epitope with evidence for epitope spreading toward CTLD1 and CTLD7 [13].

### Statistical analyses

Statistical analyses were performed using R statistical software (version 4.2.3; R Software for Statistical Computing, Vienna, Austria) and IBM SPSS Statistics (version 26; IBM Corp., Armonk, NY, USA). To decrease the variability of data, PLA2R and domain-specific IgG/G4 titers were logarithmically transformed based on the natural logarithm, and data following a non-normal distribution are presented as medians (interquartile ranges [IQRs]). Regarding data following a normal distribution, quantitative data were expressed as mean±SDs. A t-test and the Mann–Whitney U-test were used to assess differences between quantitative data, which were expressed as mean values (percentages). Finally, a chi-squared test or one-way variation analysis (ANOVA) was used to assess the differences. All probabilities were two-sided and *P*<0.05 was considered statistically significant. Logistic regression analyses were performed to confirm potential risk or protection factors of treatment responses. Variables with *P* <0.25 were chosen to be included in the binary logistic regression model. This model was constructed by using backward elimination. The nomogram and bootstrap receiver operating characteristic (ROC) curve were drawn using the R packages “rms” and”pROC,” respectively.

## Results

### Patients

The 87 patients in our cohort exhibited positive glomerular staining of the PLA2R and/or positive serum PLA2R.The predominant IgG subclass was IgG4.There were 56 male patients and 31 female patients, with a median age of 55 (41,64) years old. The level of proteinuria was 4.19(3.87,4.70)g/24 h, serum albumin was 21.79±3.63g/L and estimated glomerular filtration rate(eGFR) was 92.10(75.50,105)mL/min/1.73m2.Among them,66(73.56%)had epitope spreading (Table 1).

**Table 1:** Clinical characteristics of patients at baseline.

Characteristics	Total (N = 87)	Rituximab (N = 40)	Cyclophosphamide (N = 47)	*P*
Male sex—no. (%)	56(64.37)	27(67.50)	29(61.70)	0.574
Age—yr	55(41,64)	52.05±14.42	53.09±11.97	0.715
Hypertension—no. (%)	45(51.72)	20(50.00)	25(53.19)	0.767
Systolic BP, mmHg	131.75±16.58	130.93±15.61	132.45±17.51	0.672
Diastolic BP, mmHg	81.74±11.21	81.10±11.65	82.28±10.92	0.628
Diabetes—no. (%)	12(13.79)	7(17.5)	5(10.64)	0.355
Hemoglobin—g/L	127.16±18.48	125.28±19.04	128.77±18.05	0.383
Total Cholesterol—mmol/L	6.89(5.94,8.57)	7.01±1.93	7(6.08,9.23)	0.265
Low-density lipoprotein—mmol/L	3.85(3,5.23)	4.12±1.53	4.17±1.59	0.884
Triglyceride—mmol/L	2.11(1.51,2.89)	1.79(1.21,2.20)	2.32(1.68,3.64)	**0.002**
Serum albumin—g/L	21.79±3.63	22.11±3.63	21.52±3.64	0.457
Serum Uric acid—mmol/L	358.59±89.91	373.39±88.46	345.99±90.14	0.158
Serum creatinine—umol/L	62.60(78,90.80)	75.8(60.35,90.80)	78.9(63.50,88.70)	0.912
Estimated Glomerular Filtration Rate	92.10(75.50,105)	92.25(74,104.95)	89.21±21.82	0.956
—ml/min/1.73m2				
Urinary protein—g/24hr	4.19(3.87,4.70)	4.15(3.80,4.41)	4.26(3.88,5.08)	0.178
PLA2R-IgG (ELISA)*—RU/mL	70.72(19.63,218.75)	58.16(16.19,138.77)	117.24(28.45,300.94)	0.059
Ln PLA2R-IgG(TRFIA)	4.72(3.03,5.63)	4.61(2.87,5.51)	4.92(3.05,5.86)	0.261
Ln PLA2R-IgG4(TRFIA)	8.54(6.39,9.29)	8.25(6.86,9.28)	8.85(6.18,9.63)	0.184
Ln PLA2R-CysR-IgG^※^	3.70±1.57	3.38±1.88	4.17(2.76,5.08)	0.107
Ln PLA2R-CysR-IgG4	6.50±2.10	6.07±1.88	6.87±2.22	0.076
Ln PLA2R-CTLD1-IgG^※^	3.18(1.63,3.95)	3.09±1.43	3.49(1.23,4.36)	0.996
Ln PLA2R-CTLD1-IgG4	3.90±2.23	3.53(2.78,6.21)	3.69±2.37	0.404
Ln PLA2R-CTLD678-IgG^※^	3.74±1.48	3.57±1.78	3.87±1.20	0.375
Ln PLA2R-CTLD678-IgG4	6.86(5.26,7.92)	5.96±2.48	7.21(6.00,7.97)	**0.024**
Epitope Spreading—no.(%)	66(73.56)	27(67.50)	39(82.98)	0.093

^×^Values are expressed as means ± SD, the interquartile range (IQR) or n (%).

*Ten missing values.

^※^Domain specific antibody levels were detected by TRFIA and transfored with logarithmic transformation based on natural logarithm.PLA2R-CysR/CTLD1/CTLD678-IgG had 2 missing values.

In total, 40 and 47 patients received RTX and CTX therapy, respectively. In general, the participants in the CTX group were more likely to have hypertriglyceridemia [2.32 (1.68, 3.64) vs. 1.79 (1.21, 2.20), P = 0.002] and had higher PLA2R-CTLD678 IgG4 levels [7.21 (6.00, 7.97) vs. 5.96 ± 2.48, *P* = 0.024],compared with the RTX group. Sex, age, hypertension, diabetes, hemoglobin, serum albumin, serum creatinine, eGFR, urinary protein,anti-PLA2R antibody levels and domains specific antibody levels except for PLA2R-CTLD678 IgG4 were comparable between the two groups *(P*> 0.05).

### Treatment responses

#### Clinical remission

**Fig 1:**
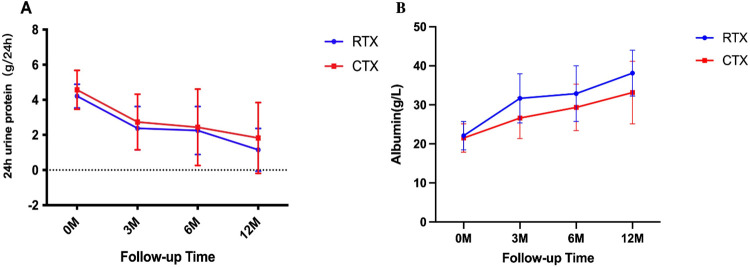
Clinical remission of the RTX and CTX groups. Temporal changes in proteinuria (A) and serum albumin (B). (Means±SD).

Clinical remission was achieved in 61/87 (70.11%) patients during 6 months of follow-up. PLA2R-IgG/-IgG4 antibody levels (*P* < 0.05) and proteinuria (*P* < 0.001) decreased in all patients regardless of treatment, especially in those who achieved remission (Table 2). The RTX and CTX groups had a similar decline in the proteinuria levels (2.27±1.37 vs. 1.86 (0.73–0.58) g/ 24 h; *P* = 0.749; Fig 1A). The clinical remission rates in the two groups were comparable (70% vs. 70.21%; *P* = 0.983; Table 2). However, a significant difference in the serum albumin levels between the two groups (32.88±7.13 vs 29.36±5.93 g/L; *P* = 0.014) was observed at the 6^th^ month (Fig 1B).

**Table 2:** Clinical characteristics of patients after 6 months treatment ^×^.

Characteristics	Remission (N = 61)	No Remission (N = 26)	*P*
Male sex—no. (%)	36(51.59)	20(76.92)	0.110
Age—yr	55(39.50,64.50)	53.38±10.95	0.974
Hypertension—no. (%)	29(47.54)	16(61.54)	0.232
Systolic BP, mmHg	131.18±16.79	133.08±16.33	0.628
Diastolic BP, mmHg	80.93±11.32	83.62±10.93	0.310
ACEIs/ARBs—no. (%)	43(70.49)	17(65.38)	0.637
Diabetes—no. (%)	9(14.75)	3(11.54)	1.000
Hemoglobin—g/L	127.28±17.78	126.88±20.40	0.928
Total Cholesterol—mmol/L	6.89(6.07,8.69)	7.13±2.10	0.663
Low-density lipoprotein—mmol/L	4.15±1.46	4.15±1.78	0.986
Triglyceride—mmol/L	1.97(1.33,3.06)	2.16(1.64,3.08)	0.366
Serum albumin—g/L	22.11±3.18	21.04±4.49	0.277
Serum Uric acid—mmol/L	366.51±96.23	339.99±71.21	0.159
Serum creatinine—umol/L	73.10(59.30,88.00)	83.55(68.30,105.63)	0.062
Estimated Glomerular Filtration Rate	92.28±20.84	87.75(67.73,103.95)	0.085
—ml/min/1.73m2			
Urinary protein—g/24hr	4.08(3.82,4.50)	4.55(4.01,5.21)	**0.016**
PLA2R-IgG(ELISA)—RU/mL	61.38(15.28,158.97)	105.80(35.87,420.92)	0.077
Ln PLA2R-IgG(TRFIA)	4.72(2.72,5.53)	4.92±1.59	0.126
Ln PLA2R-IgG4(TRFIA)	8.47(5.87,9.27)	8.88(7.89,9.66)	0.061
Ln PLA2R-CysR-IgG	3.84(2.47,4.62)	3.90±1.91	0.623
Ln PLA2R-CysR-IgG4	6.25±2.08	7.11±2.07	0.080
Ln PLA2R-CTLD1-IgG	3.14(1.44,3.97)	3.12±1.49	0.590
Ln PLA2R-CTLD1-IgG4	3.55±2.18	4.71±2.19	**0.026**
Ln PLA2R-CTLD678-IgG	3.86(2.76,4.51)	4.16±1.66	0.145
Ln PLA2R-CTLD678-IgG4	6.38±2.13	7.41(5.97,8.61)	0.106
Epitope Spreading—no.(%)	46(75.41)	20(76.92)	0.880
Urinary protein M6—g/24hr	1.40(0.65,2.26)	4.12(3.58,4.68)	**<0.001**
Serum albumin M6—g/L	33.50±5.15	25.20(21.18,27.50)	**<0.001**
Serum creatinine M6—umol/L	69.30(59.55,82.55)	79.85(65.10,103.28)	**0.019**
PLA2R-IgG(ELISA)*—RU/mL	2.01(1.42,9.17)	16.28(4.19,133.37)	**<0.001**
Ln PLA2R-IgG(TRFIA) M6^※^	1.87(1.34,4.24)	3.59±1.79	**0.028**
Ln PLA2R-IgG4(TRFIA) M6^※^	3.63(2.37,7.41)	6.72±2.72	**0.016**
Ln PLA2R-CysR-IgG M6^¥^	2.40±1.72	3.14±1.78	0.184
Ln PLA2R-CysR-IgG4 M6*	3.10(2.23,5.18)	5.64±2.81	**0.021**
Ln PLA2R-CTLD1-IgG M6^#^	-0.06(-0.69,3.04)	2.40±2.20	0.054
Ln PLA2R-CTLD1-IgG4 M6*	1.79±1.81	3.15±2.58	**0.030**
Ln PLA2R-CTLD678-IgG M6^#^	2.28±1.51	2.65±1.47	0.443
Ln PLA2R-CTLD678-IgG4 M6*	2.76±2.28	5.03±2.23	**0.001**
Epitope Spreading M6—no.(%)	8(24.24)	10(52.63)	**0.038**
Treatment			0.983
RTX	28(45.90)	12(46.15)	
CTX	33(54.10)	14(53.85)	

^×^Values are expressed as means ± SD, the interquartile range (IQR) or n (%).Domain specific antibody levels were detected by TRFIA and transfored with logarithmic transformation based on natural logarithm.

*7 missing values.

^※^26 missing values.

^¥^ 36 missing values.

^#^35 missing values.

*28 missing values.

#### Immunological remission

In the RTX group, 27 (69.23%) patients tested positive for the PLA2R-IgG (ELISA) at baseline, with a median level of 58.16 (16.19, 138.77) RU/mL. As to the CTX group, there were 31 (81.58%) serum-positive patients, with a median level of 117.24 (28.45,300.94).After 6 months of RTX therapy, 28 of 40 (70%) patients achieved clinical remission in the RTX group, while 3/14 (21.43%) patients achieved immunological remission with PLA2R-IgG4 depletion (<66.66 RU/mL). In the CTX group, 33/47 (70.21%) patients achieved clinical remission, and 13 (61.90%) patients achieved immunological remission with PLA2R-IgG4 antibody depletion (<66.66 RU/mL). A significant difference in the PLA2R-IgG4 depletion rate was observed between the two treatment groups, detected using the TRFIA assay (*P* = 0.019; Table 3).

**Table 3:** Clinical and immunological response of patients at month 6.

	RTX	CTX	*P*
Proteinuria remission rate	28/40(70)	33/47(70.21)	0.983
PLA2R-IgG depletion rate-ELISA	13/26(50)	16/24(66.67)	0.233
PLA2R-IgG depletion rate -TRFIA	3/14(21.43)	11/21(52.38)	0.067
PLA2R-IgG4 depletion rate-TRFIA	3/14(21.43)	13/21(61.90)	**0.019**
PLA2R-CysR-IgG4 depletion rate	5/14(35.71)	7/19(36.84)	0.947
PLA2R-CTLD1-IgG4 depletion rate	6/14(42.86)	5/19(26.32)	0.459
PLA2R-CTLD678-IgG4 depletion rate	6/14(35.71)	12/19(63.16)	0.247
Epitope spreading reversion rate	6/14(42.86)	8/19(42.11)	0.966

Epitope spreading was observed in 27/40 (67.50%) RTX patients and 39/47 (82.98%) CTX patients at baseline. After six months of treatment, an observed difference in epitope spreading between patients who achieved remission and those who did not (*P* = 0.023) was noted specifically in the CTX group (Table 4). Furthermore, the PLA2R-CysR-IgG4 (*P* = 0.030), PLA2R-CTLD1-IgG4 (*P* = 0.005), and PLA2R-CTLD678-IgG4 (*P* = 0.003) levels showed significant differences (Table 4). In the entire study cohort, significant differences were observed in PLA2R-CysR-IgG4 (*P* = 0.021), PLA2R-CTLD1-IgG4 (*P* = 0.030), PLA2R-CTLD678-IgG4 (*P* = 0.001), and epitope spreading (*P* = 0.038) (Table 2).

**Table 4:** Clinical characteristics of patients of different treatments after 6 months.

Characteristics	Remission	No Remission	*P*
**Rituximab(N = 40)**			
Male sex—no. (%)	18(64.29)	9(75)	0.716
Age—yr	50.64±15.45	55.33±11.57	0.352
Hypertension—no. (%)	13(46.43)	7(58.33)	0.490
Systolic BP, mmHg	131.96±15.41	128.50±16.48	0.527
Diastolic BP, mmHg	81.07±11.52	81.17±12.47	0.981
Diabetes—no. (%)	5(17.86)	2(16.67)	1.000
Hemoglobin—g/L	126.61±17.35	122.17±23.06	0.506
Total Cholesterol—mmol/L	7.17±1.67	6.15(5.06,6.99)	0.148
Low-density lipoprotein	4.30±1.37	3.70±1.83	0.260
Triglyceride	1.60(1.12,2.00)	2.12(1.50,2.77)	0.057
Serum albumin—g/L	22.08±3.03	22.18±4.91	0.944
Serum Uric acid, mmol/L	381.85±95.61	353.66±68.58	0.362
Serum creatinine—umol/L	73.05(59.03,90.45)	81.95(66,145.18)	0.275
Estimated Glomerular Filtration Rate	93.81±20.13	75.01±31.28	0.028
—ml/min/1.73m2			
Urinary protein—g/24hr	4.04(3.76,4.33)	4.50±0.66	0.067
PLA2R-IgG(ELISA)—RU/mL*	43.37(13.08,76.47)	99.61(22.70,389.36)	**0.046**
Ln PLA2R-IgG(TRFIA)	4.67(2.80,5.35)	4.77±2.00	0.299
Ln PLA2R-IgG4(TRFIA)	8.06(6.86,9.05)	7.89±2.32	0.389
Ln PLA2R-CysR-IgG^※^	3.20±1.60	3.76±2.42	0.480
Ln PLA2R-CysR-IgG4	5.92±1.78	6.42±2.14	0.455
Ln PLA2R-CTLD1-IgG^※^	3.12±1.40	3.05±1.54	0.896
Ln PLA2R-CTLD1-IgG4	3.97±2.01	4.55±2.23	0.420
Ln PLA2R-CTLD678-IgG^※^	3.98(2.42,4.44)	4.05±2.11	0.470
Ln PLA2R-CTLD678-IgG4	6.14±1.96	5.53±3.49	0.575
Epitope Spreading M0—no.(%)	20(71.43)	7(58.33)	0.476
Urinary protein M6—g/24hr	1.58±0.99	3.88±0.53	**<0.001**
Serum albumin M6—g/L	35.08±6.10	26.15(23.28,29.25)	**0.001**
Serum creatinine M6—umol/L	70.80(61.23,87.68)	79.85(64.78,101.70)	0.268
PLA2R-IgG(ELISA) M6—RU/mL^#^	2.35(1.38,9.44)	11.64(2.98,57.46)	**0.029**
Ln PLA2R-IgG(TRFIA) M6^¥^	2.32±1.88	2.70±1.83	0.647
Ln PLA2R-IgG4(TRFIA) M6^¥^	5.08±2.38	5.09±2.80	0.997
Ln PLA2R-CysR-IgG M6*	1.84±1.72	2.50±1.83	0.536
Ln PLA2R-CysR-IgG4 M6^¥^	3.58±1.81	4.44±3.12	0.418
Ln PLA2R-CTLD1-IgG M6*	2.58±0.74	3.20±0.86	0.186
Ln PLA2R-CTLD1-IgG4 M6^¥^	1.48(1.00,2.61)	1.71±1.50	0.891
Ln PLA2R-CTLD678-IgG M6*	2.48±2.36	3.21±0.45	0.448
Ln PLA2R-CTLD678-IgG4 M6^¥^	2.90±1.88	4.20±2.11	0.153
Epitope Spreading M6—no.(%)	3(21.43)	2(25)	1.000
**Cyclophosphamide(N = 47)**			
Male sex—no. (%)	18(54.55)	11(78.57)	0.121
Age—yr	53.67±12.64	51.71±10.52	0.614
Hypertension—no. (%)	16(48.48)	9(64.29)	0.321
Systolic BP, mmHg	130.52±18.09	137±15.72	0.250
Diastolic BP, mmHg	80.82±11.32	85.71±9.38	0.162
Diabetes—no. (%)	4(12.12)	1(7.14)	1.000
Hemoglobin—g/L	127.85±18.40	130.93±17.67	0.598
Total Cholesterol—mmol/L	6.89(5.98,9.20)	7.55±1.69	0.593
Low-density lipoprotein	4.02±1.54	4.52±1.71	0.329
Triglyceride	2.38(1.70,3.56)	2.22(1.63,3.65)	0.675
Serum albumin—g/L	22.14±3.35	20.06±4.01	0.073
Serum Uric acid, mmol/L	353.50±96.28	328.27±73.83	0.386
Serum creatinine—umol/L	76.02±21.37	83.55(68.30,96.55)	0.146
Estimated Glomerular Filtration Rate	90.99±21.65	85.02±22.44	0.397
—ml/min/1.73m2			
Urinary protein—g/24hr	4.33±0.73	4.70(4.00,5.60)	0.096
PLA2R-IgG(ELISA)—RU/mL^**§**^	117.50(23.16,287.29)	115.87(41.72,829.26)	0.361
Ln PLA2R-IgG(TRFIA)	4.86(2.67,5.84)	5.05±1.20	0.340
Ln PLA2R-IgG4(TRFIA)	8.51(5.67,9.57)	8.95±1.09	0.108
Ln PLA2R-CysR-IgG	4.16(2.70,5.04)	4.02±1.42	0.693
Ln PLA2R-CysR-IgG4	6.52±2.29	7.70±1.88	0.097
Ln PLA2R-CTLD1-IgG	3.50(-0.69,4.32)	3.17±1.50	0.441
Ln PLA2R-CTLD1-IgG4	3.20±2.28	4.84±2.23	**0.028**
Ln PLA2R-CTLD678-IgG	3.71±1.18	4.26±1.22	0.155
Ln PLA2R-CTLD678-IgG4	6.93(5.65,7.69)	8.03±1.61	**0.036**
Epitope Spreading M0—no.(%)	26(78.79)	13(92.86)	0.405
Urinary protein M6—g/24hr	1.01(0.53,2.02)	4.38(3.69,6.11)	**<0.001**
Serum albumin M6—g/L	32.16±3.77	22.75±4.77	**<0.001**
Serum creatinine M6—umol/L	67.20(57.30,78.20)	78.9(65.1,104.18)	**0.038**
PLA2R-IgG(ELISA) M6—RU/mL^ɭ^	1.94(1.45,9.24)	31.54(8.66,145.16)	**0.001**
Ln PLA2R-IgG(TRFIA) M6^&^	1.80(1.35,4.27)	4.23±1.53	**0.009**
Ln PLA2R-IgG4(TRFIA) M6^&^	3.35(1.74,7.09)	8.19(7.33,8.90)	**0.006**
Ln PLA2R-CysR-IgG M6^£^	2.57±1.72	3.48±1.74	0.175
Ln PLA2R-CysR-IgG4 M6^£^	3.10(2.35,5.72)	6.50±2.32	**0.030**
Ln PLA2R-CTLD1-IgG M6^£^	-0.69(-0.69,2.19)	1.96±2.60	0.091
Ln PLA2R-CTLD1-IgG4 M6^£^	1.58±1.95	4.20±2.75	**0.005**
Ln PLA2R-CTLD678-IgG M6^£^	2.21±1.13	2.33±1.75	0.815
Ln PLA2R-CTLD678-IgG4 M6^£^	2.65±2.58	5.65±2.21	**0.003**
Epitope Spreading M6—no.(%)	5(26.32)	8(72.73)	**0.023**

^×^Values are expressed as means ± SD, the interquartile range (IQR) or n (%).Domain specific antibody levels were detected by TRFIA and transfored with logarithmic transformation based on natural logarithm.

*1 missing value.

^※^2 missing values.

^#^4 missing values.

^¥^18 missing values.

*PLA2R-CysRIgG had 28 missing values.PLA2R-CTLD1/678-IgG had 27 missing values.

^**§**^ 9 missing values.

^ɭ^8 missing values.

^&^15 missing values.

^£^17 missing values.

### Correlation of PLA2R domain-specific IgG/IgG4 TRFIA to the total PLA2R-IgG/IgG4

**Fig 2:**
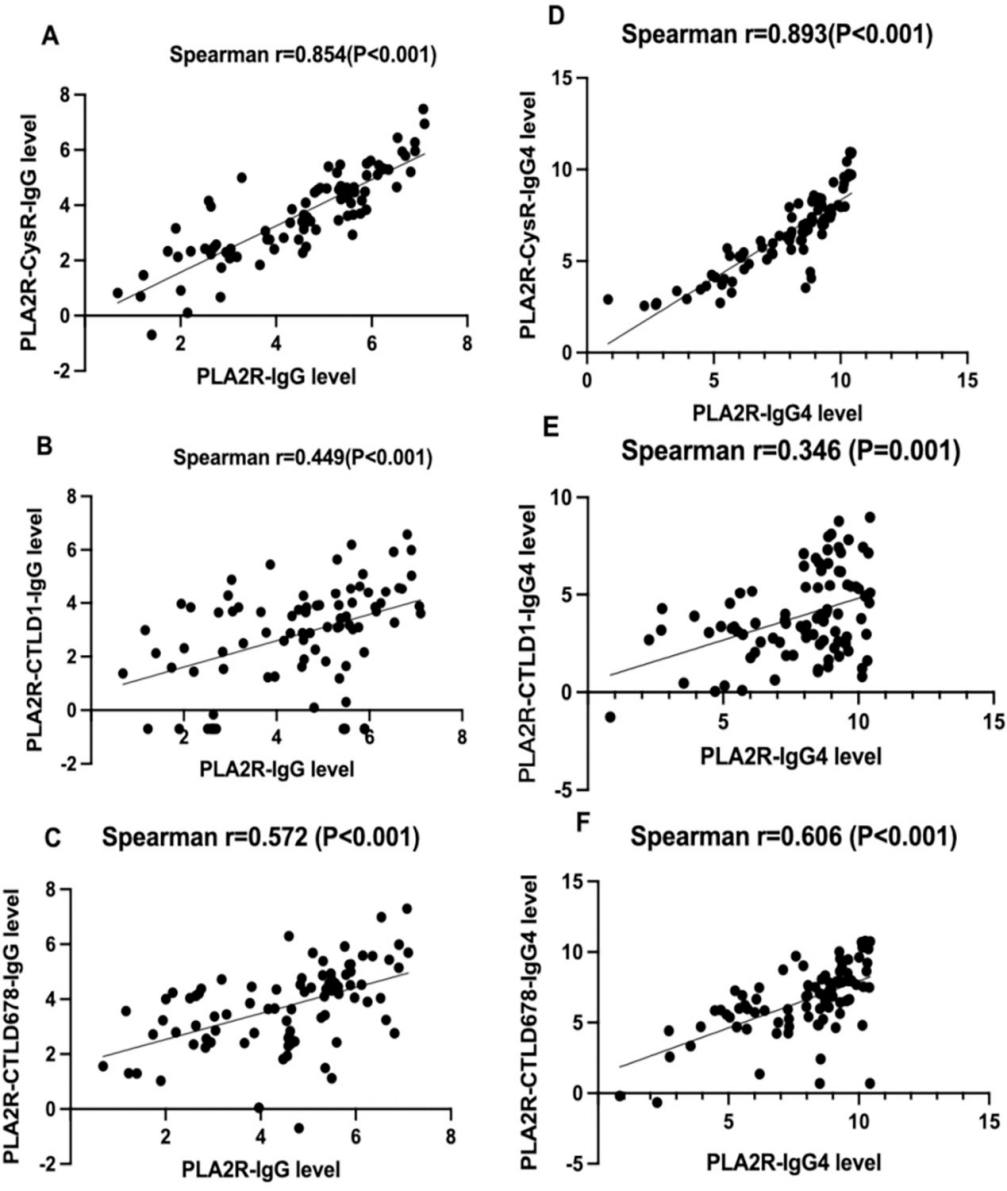
Correlation analysis between domain-specific antibody levels and PLA2R antibody levels.

We set up three TRFIAs to specifically measure IgG and IgG4 antibodies targeting CysR, CTLD1, and CTLD678. In addition, the correlation between the domain-specific antibody levels and PLA2R antibody levels detected by IgG and IgG4 was calculated. As shown in Fig 2A–2C, the Spearman’s correlations of PLA2R-IgG level with PLA2R-CysR-IgG,CTLD1 IgG and CTLD678-IgG levels were 0.854,0.449 and 0.572,respectively.The Spearman’s correlations of PLA2R-IgG4 level with PLA2R-CysR-IgG4,CTLD1-IgG4 and CTLD678-IgG4 levels were 0.893,0.346 and 0.606,respectively (Fig 2D–2F). Domain specific antibody levels were strongly associated with the total anti-PLA2R antibody levels.

### Binary logistic regression analysis of proteinuria remission factors

**Fig 3:**
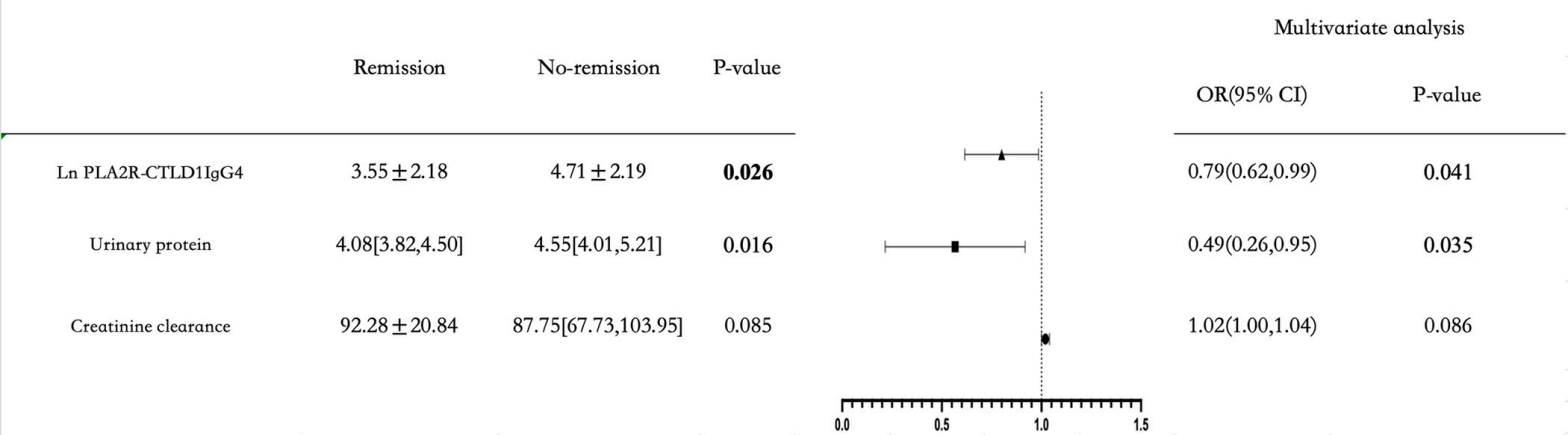
Forrest plot of multivariate logistic regression.

Compared to the responders, the non-responders were more likely have higher levels of urinary protein [4.55 (4.01,5.21) vs.4.08 (3.82,4.50),*P* = 0.016], PLA2R-CTLD1-IgG4 [4.71 ± 2.19 vs .3.55± 2.18,*P* = 0.026] at baseline (Table 2). The univariate logistic regression showed that creatinine clearance (odds ratio(OR): 1.02, 95% confidence interval (CI): 1.00–1.04,*P* = 0.034), urinary protein (OR: 0.49, 95%CI: 0.27–0.88,*P* = 0.016) and PLA2R-CTLD1-IgG4 (OR: 0.79,95%CI: 0.63–0.98,*P* = 0.030) were associated with proteinuria remission at the 6^th^ month (Table 5). Multivariate logistic regression analysis showed that the higher level of urinary protein (OR: 0.49,95%CI: 0.26–0.95,*P* = 0.035) and PLA2R-CTLD1-IgG4 (OR: 0.79,95%CI: 0.62–0.99,*P* = 0.041) were independent risk factors for disease early remission (Fig 3).

**Table 5:** Binary logistic regression analysis of proteinuria remission factors.

	Univariate analysis		Multivariate analysis	
	OR(95% CI)	P-value	OR(95% CI)	P-value
Sex(Male)	2.32(0.81,6.58)	0.116		
Serum Uric acid	1.00(0.99,1.01)	0.209		
Creatinine clearance	1.02(1.00,1.04)	**0.034**	1.02(1.00,1.04)	0.086
Urinary protein	0.49(0.27,0.88)	**0.016**	0.49(0.26,0.95)	**0.035**
PLA2R-IgG(ELISA)	0.99(0.99,1.00)	0.063		
PLA2R-IgG(TRFIA)	0.77(0.57,1.05)	0.093		
PLA2R-IgG4(TRFIA)	0.80(0.62,1.04)	0.092		
PLA2R-CysR-IgG4	0.82(0.65,1.03)	0.084		
PLA2R-CTLD1-IgG4	0.79(0.63,0.98)	**0.030**	0.79(0.62,0.99)	**0.041**
PLA2R-CTLD678-IgG	0.74(0.53,1.04)	0.082		
PLA2R-CTLD678-IgG4	0.91(0.74,1.12)	0.376		

### Development of an individualized prediction model

**Fig 4:**
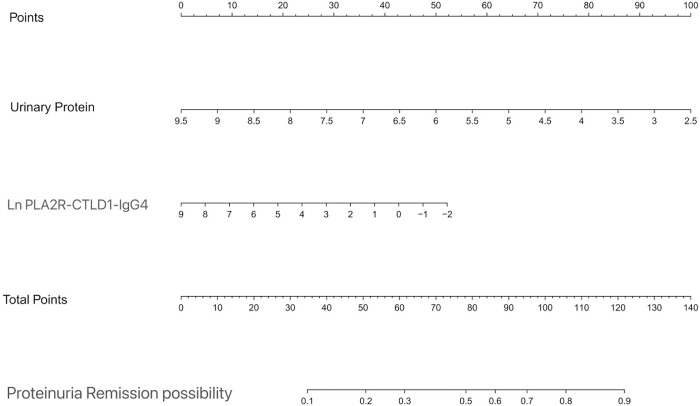
Nomogram predicting proteinuria remission at the 6th month.

**Fig 5:**
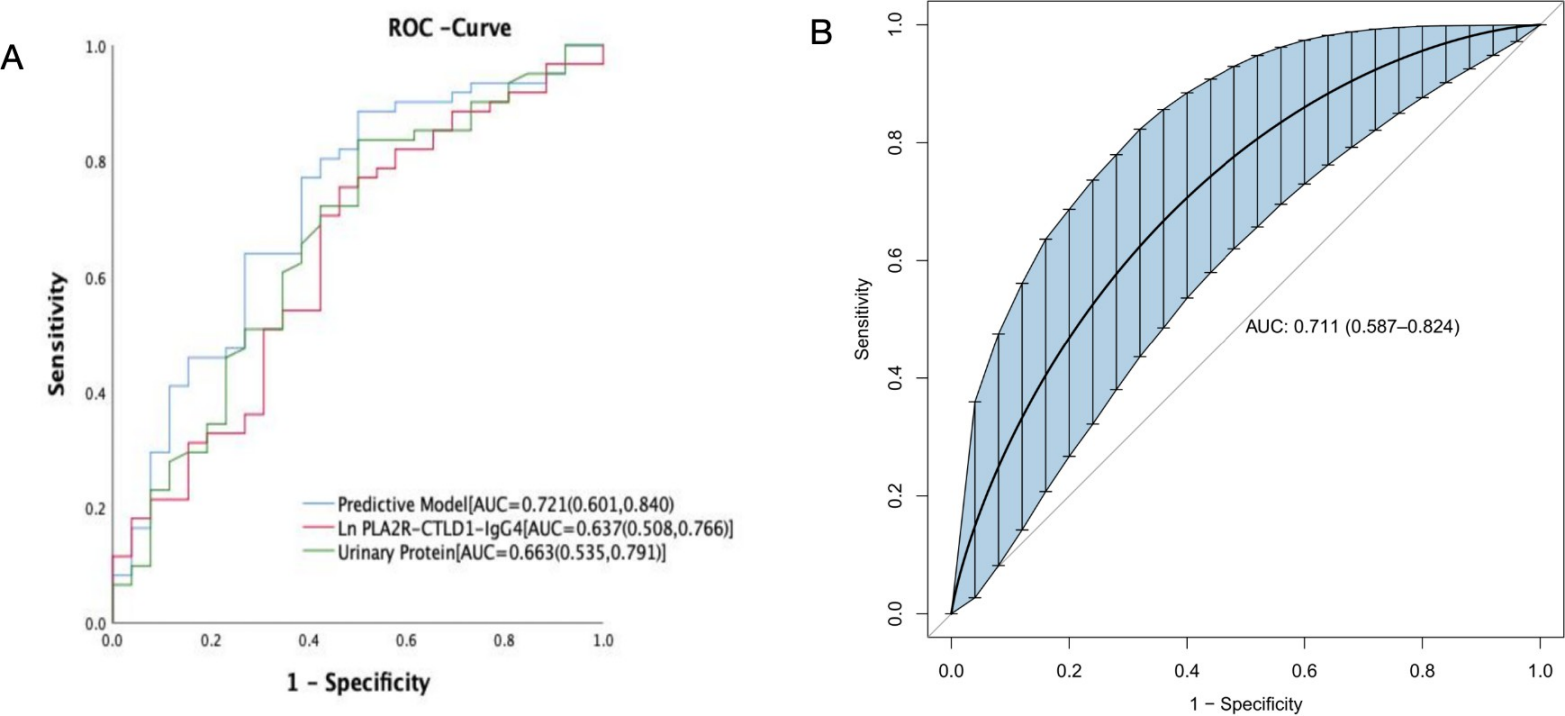
ROC curve (A) and bootstrape ROC curve (B) analysis of prediction model for proteinuria remission at the 6th month.

Multivariate logistic regression analysis demonstrated that two variables urinary protein and PLA2R-CTLD1-IgG4 were independently associated with disease early remission. A model incorporating the two potential predictors is presented as a nomogram (Fig 4). The nomogram was assigned a specific score, and the total score was used to obtain the probability of disease early remission possibility. The ratios of the calculated beta were used to evaluate the proportional predictive effects of these variables. The projections from total points on the scales below indicated the estimated probability of proteinuria remission. Therefore, the best prediction model we proposed was as follows: PMN early remission possibility = 5.055–0.721urinary protein-0.239PLA2R-CTLD1IgG4 level. The area under the receiver operating characteristic curve (AUC-ROC) in our model is 0.721 (95% CI, 0.601,0.840) (Fig 5A).

The nomogram was developed in the entire cohort based on urinary protein and PLA2R-IgG4 level. Points for each variable were acquired by drawing a straight line upward from the corresponding value to the ‘Points’ line. Then sum the points received from each variable and locate the number on the ‘Total Points’ axis. To conclude the patient’s sort probability of proteinuria remission at the 6th month, draw a straight line down to the corresponding ‘Proteinuria Remission Possibility’ axis.

### Internal validation of the model

Given the small sample size, we used the bootstrap method to validate our prediction model. After 1000 repeated samplings, the AUC-ROC is 0.711 (95% CI: 0.587–0.824) (Fig 5B).

## Discussion

MN is an autoimmune disease in which autoantibodies combine with antigens expressed on the glomerular podocytes. The 2021 KDIGO guideline recommends RTX as the first-line therapy, while CTX remains the preferred option for very high-risk cases [10]. There is no precise evidence for the treatment of moderate and high risk PMN patients. This retrospective study enrolled 87 patients who was diagnosed with PLA2R-associated PMN from 2020 to 2022 with a moderate or high risk of progressive loss of kidney function and compared the effectiveness of RTX and CTX regimen in those patients.

Both RTX and CTX therapy showed good efficacy in achieving clinical remission in PMN patients(70%vs.70.21%,*P* = 0.983).When RTX was administrated as first-line therapy, patients’ clinical remission(70%) was comparable to the remission rate of 69.1% reported by Ruggenenti et al. [24], and even higher than those reported in the MENTOR [23] and GEMRITUX study [14]. (60.0% and 64.9%, respectively)

The clinical remission rate for CTX therapy was 70.21%, which was similar to rates reported in other observational studies. A retrospective analysis of 32 cases with PMN treated with intravenous CTX (500–750 mg/m2 every month for 6 months) plus steroids reported that 81% of the patients achieved clinical remission [25]. A long-term observational study of 55 PMN patients found that 70.9% of patients achieved clinical response with CTX regimen [26]. Another retrospective analysis studied 40 patients treated with intravenous CTX (600 mg/m2 every 4 weeks for up to 6 months) plus steroids. In this study, 67% of patients reached some form of remission [27]. However, the RTX regimen could induce more rapid normalization of serum albumin compared with the CTX regimen. Supported by the GEMRITUX study, the serum albumin increase preceded that of proteinuria [14]. RTX directly targets CD20-B cells to reduce the production of circulating antibodies and prevent the formation of immune deposits thus maintaining the integrity of podocytes [28]. In addition, it has a potential effect on T-cells, including reducing the release of cytokines (e.g., interleukin-13,) helping recover the function of regulatory T-cells, and stabilizing the cytoskeleton [29, 30].

Consistent with the RI-CYCLO study [31], our study also indicated that the CTX regimen may induce immunological remission earlier than the RTX regimen at the 6-months observation. The aforementioned findings were supported by those of a small study conducted in the United Kingdom that included new-incident and relapse patients, the anti-PLA2R levels decreased from 244 U/L to <14 U/L at 6 months, and 44% of the new-incident patients achieved complete response after a median follow-up period of 32 months [32]. CTX induces a generalized leukocyte and mature plasma cell ablation, resulting in a more drastic reduction in antibody production than RTX which is a more specifically targeted drugs. Early immunological response appears to be a strong predictor of clinical remission [4, 6, 33–35]. Stefan et al reported that the negativity of anti-PLA2R antibodies at 3 months after diagnosis was associated with a 60% increase in the chances of later remission [33]. In a study by Ruggenenti et al., a 50% reduction in anti-PLA2R titer preceded an equivalent reduction in proteinuria by 10 months [34]. However, there were still other studies found only a weak or no relationship between the remission rate and anti-PLA2R antibodies titer [2, 36–38]. Moreover, the high sensitivity of PLA2R-IgG4 should be considered in the future disease monitoring instead of total PLA2R-IgG level. In our previous study, we have quantitatively detected PLA2R-IgG4 antibody level and confirmed their clinical values in risk stratification and treatment effectiveness analysis [20, 21].

The discrepancies between the clinical outcomes and anti-PLA2R titers have motivated researchers to find other biomarkers to assist in the evaluation of disease prognosis. Three important studies have shown that at least three PLA2R epitope regions are targeted by autoantibodies in MN [11–13]. In a study conducted by Seitz-Polski et al, they stratified patients positive for PLA2R by serum reactivity to one or more of these domains and reported that epitope spreading beyond the CysR epitope was an independent risk factors of poor renal prognosis [12]. Whether the level of antibodies specific to a certain PLA2R epitope region may have a better prediction value than anti-PLA2R level is worth researching. To address this question we used TRFIA assay to quantify the titer of the individual PLA2R domain-specific antibody. Using multivariate logistic analysis, we discovered that it was PLA2R-CTLD1-IgG4 antibody level(*P* = 0.041) be independently associated with disease proteinuria remission at the 6^th^ month instead of PLA2R-IgG(*P* = 0.063).As with the GEMRITUX study [13],we have also found that epitope spreading beyond CysR domain at baseline was associated with a decreased remission rate at month 6.The question of why PLA2R-CTLD1-IgG4 holds the potential to be a new biomarker for predicting disease remission to be addressed. PMN is an autoimmune disease, and in most cases antibodies typically first recognize the immunodominant epitope (CysR for PLA2R). When epitope spreading occurs, it initially extends to non-cross reactive epitopes on the same protein(CTLD1 for PLA2R). Interestingly, one patient, whose epitope reactivity was negative at baseline and became an epitope spreader at the 6^th^ month, suffered from persistent nephrotic syndrome and treatment resistance, despite a relatively low anti-PLA2R antibody titer. This may explain the discrepancies observed between clinical features and the anti-PLA2R titers, which suggests that the domain-specific antibody level can be a more potent predictor of disease remission than the PLA2R antibody titer.

Furthermore, we established an individualized model to predict disease proteinuria remission possibilities at 6 months. In our study, two independent risk factors (i.e., proteinuria and PLA2R-CTLD1-IgG4) were used to construct an easy-to-use nomogram model. The predictive ability of a new prognostic score is critical for decision making regarding treatment strategies and for predicting outcomes in patients with PMN. The nomogram we constructed showed superior predictive ability (all AUC-ROCs were > 0.70) in both the training cohorts and internal validation. Thus, the nomograms we have established are accurate, widely beneficial and user-friendly in clinical practice. For example, the total score of a patient diagnosed with PLA2R-associated PMN with 24h urine protein of 4.5 g and LnPLA2R-CTLD1-IgG4 of 3.97 is 94. Therefore the probability of early proteinuria remission is 70%. There is a possibility of delayed immunosuppression therapy for this patient. Under these circumstances, our established nomogram might be utilized as a more powerful and conventional tool to predict disease proteinuria remission and help the decision of early therapeutic intervention.

This study had some limitations. First, it was a retrospective, single-center study with a small sample size, limiting the significance of the statistical analysis of therapeutic evaluation. Additionally, many values of PLA2R-CysR/CTLD1/CTLD678-IgG/-IgG4 were missing because of the retrospective nature of the study. Second, the endpoint of our study was the remission rate of proteinuria after immunosuppressive therapy for 6 months, and the follow-up time should be prolonged because RTX may require a longer time to respond, as indicated by previous studies. More prospective multi-center studies are needed to validate our findings.

## Conclusions

In conclusion, the CTX regimen can induce immunological remission earlier than RTX regimen at the span of 6 months, while the overall clinical remission rate is comparable between regimes. The PLA2R-CTLD1-IgG4 level has a better predict value at proteinuria remission at the 6^th^ month than total anti-PLA2R-IgG antibody level.

## Supporting information

S1 File Raw data. (XLSX)
